# A Rare Case Report of Wunderlich Syndrome in a Chronic Hemodialysis Patient

**DOI:** 10.3390/reports8030121

**Published:** 2025-07-25

**Authors:** Elizabeth Artinyan, Evelina Valcheva, Marina Vaysilova, Nikolay Dimov

**Affiliations:** 1 Nephrology Clinic, University Hospital “Sv. Georgi” Plovdiv, 4000 Plovdiv, Bulgaria; evelinavalcheva98@gmail.com (E.V.); vaiss@abv.bg (M.V.); nikolai.r.dimov@gmail.com (N.D.); 2 Second Department of Internal Diseases, Section of Nephrology, Medical University Plovdiv Bulgaria, 4000 Plovdiv, Bulgaria

**Keywords:** Wunderlich syndrome, spontaneous renal hematoma, hemodialysis

## Abstract

**Background and Clinical Significance:** Spontaneous renal hematoma, also known as Wunderlich syndrome (WS), is a rare disease characterized by the acute onset of spontaneous renal hemorrhage into the subcapsular, perirenal, and/or pararenal spaces without a history of prior trauma. WS can be a life-threatening condition due to hemorrhagic shock; consequently, prompt diagnosis and a therapeutic approach are essential for favorable outcomes. Treatment ranges from conservative management to surgical intervention. The most common etiologies are neoplasms and vascular diseases, but WS can also be observed in patients undergoing hemodialysis. In patients with end-stage renal disease (ESRD), especially those on hemodialysis, acquired cystic kidney disease and renal cell carcinoma are among the primary causes of WS. Although less common, WS can develop in dialysis patients even in the absence of traditional (primary) risk factors. In general, patients with chronic kidney disease (CKD) have a paradoxical hemostatic profile, likely explaining their higher tendency to bleed, so WS can occur without existing predisposing factors. The multifactorial pathogenesis in these patients includes functional platelet abnormalities, intimal arterial fibrosis, chronic inflammation, and oxidative stress associated with ESRD. The use of hemodialysis-related antithrombotic medications could serve as another contributing factor increasing the risk of bleeding. **Case Presentation:** We present a case report of a 62-year-old male on chronic dialysis who developed sudden right-sided lumbar pain and hematuria during dialysis without evidence of prior trauma. Imaging revealed a large subcapsular hematoma of the right kidney. Further investigations did not reveal additional risk factors in this instance; however, his routinely used hemodialysis-related antithrombotic medications were potentially a contributing factor. Despite conservative treatment, his condition worsened, and the hematoma enlarged, requiring emergency nephrectomy. Postoperatively, his condition gradually improved. **Conclusions:** This case highlights the importance of considering WS in hemodialysis patients, even without the presence of traditional risk factors, as well as including WS in the differential diagnosis of acute abdominal pain.

## 1. Introduction and Clinical Significance

Wunderlich syndrome (WS) is a rare clinical syndrome characterized by the acute onset of spontaneous renal hemorrhage into the subcapsular, perirenal, and/or pararenal spaces without a history of prior trauma [1]. The most common etiologies are renal neoplasms and vascular disease, while rarer causes of WS include renal infections, cystic disease, coagulation disorders, and the use of anticoagulation therapy [1]. Despite being rare, spontaneous renal hematoma (SRH) may also occur in patients with chronic kidney disease (CKD), especially those who undergo hemodialysis treatment. The reported prevalence in patients on dialysis is less than 1% in a 10-year period; however, the exact incidence remains unknown [2]. In patients with end-stage renal disease (ESRD), WS is traditionally associated with acquired cystic kidney disease (ACKD) or renal cell carcinoma (RCC). Additionally, antithrombotic agents used for hemodialysis (HD) and CKD-related hemostasis abnormalities probably act as contributing factors [3]. We present a case of a 62-year-old male with ESRD on chronic hemodialysis who developed Wunderlich syndrome without the main recognizable risk factors.

## 2. Case Presentation

This case study follows a 62-year-old male with a history of moderate arterial hypertension and no other comorbidities (including kidney diseases) up to 2019, when severe azotemia was detected while abdominal ultrasonography revealed bilateral nephrosclerosis. Since then, he has been undergoing regular hemodialysis treatment via arteriovenous fistula (AVF). His systemic outpatient therapy consisted of antihypertensive therapy—amlodipine 10 mg q.d. and bisoprolol 5 mg q.d.—and dialysis-related therapy—acetylsalicylic acid 100 mg q.d., erythropoietin supplementation, and the administration of 0.4 mL of nadroparin alongside dialysis procedures.

**Figure 1:**
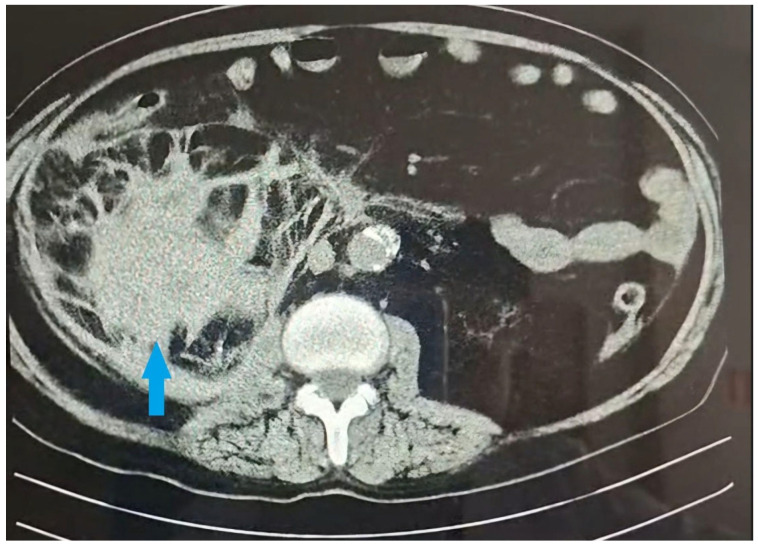
Contrast-enhanced CT in the scan-axial plane. The blue arrow indicates the massive subcapsular hematoma of the right kidney (size 10.8 × 8.6 cm).

During a regular dialysis session, he complained of severe pain in his right lumbar region and hematuria. A detailed medical history revealed that the patient experienced mild right-sided lumbar tenderness 9–10 h prior to the current dialysis procedure while engaging in routine daily activities. He denied any preceding trauma. On physical examination, he presented afebrile, with pale, sweaty skin and arterial hypotension (BP 90/60 mmHg). A bedside abdominal ultrasound demonstrated subcapsular free fluid of the right kidney. Emergency contrast-enhanced computed tomography (CT) confirmed a massive subcapsular hematoma of the right kidney (size 10.8 × 8.6 cm) (Figure 1), infiltrating the retroperitoneal fat tissue. The patient was admitted to the urology clinic.

**Figure 2:**
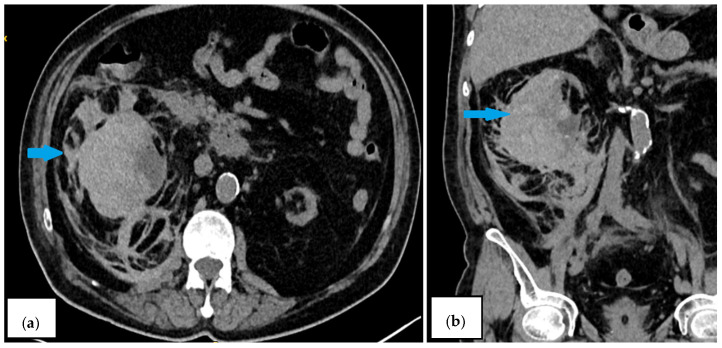
Contrast-enhanced CT scan on the second day ((**a**)—axial plane; (**b**)—coronal plane). The blue arrows indicate an increase in hematoma size to 12.37 × 6.23 cm and the presence of retroperitoneal blood collection.

Laboratory tests revealed a decrease in hemoglobin levels to 78 g/L (a drop of 40 g/L compared with previous results a few weeks prior); normal coagulation tests; no thrombocytopenia; and unremarkable results for other laboratory tests, with the exception of azotemia. Conservative therapy with blood transfusions, antibiotics, hemostatic agents, and analgesics was initiated. On his second day of hospitalization, his laboratory results indicated worsening anemia, and a follow-up abdominal CT scan showed an increase in hematoma size and the presence of retroperitoneal blood collection (Figure 2a,b). Emergency open surgery was performed. Intraoperatively, a transverse lesion was identified in the middle of the right kidney. The right kidney was removed, and the whole specimen was sent for pathological examination. The histology findings confirmed a nephrosclerotic kidney with extensive subcapsular parenchymal hemorrhages and marked chronic tubulointerstitial nephritis. In the following days, the patient continued his treatment with blood transfusions, leading to improvement in and normalization of his hematologic parameters.

## 3. Discussion

Renal hematomas are relatively rare but clinically significant conditions. The primary causes of renal hematomas can be categorized as traumatic, spontaneous, or iatrogenic, with a retrospective review from 2011 to 2021 reporting the distribution as 21%, 32%, and 47%, respectively [4]. Spontaneous renal hematoma, also known as Wunderlich syndrome, was first described by Bonet in 1679 and later by Wunderlich [1]. A meta-analysis of SRH cases from 1985 to 1999 identified 165 cases, while a recent systematic review identified an additional 102 cases [5].

In general, renal neoplasms—most commonly, benign angiomyolipomas and renal cell carcinoma (RCC)—account for 60–65% of cases, while vascular diseases (such as vasculitis, renal artery aneurysms, arteriovenous malformations, and renal vein thrombosis) represent the second most common cause, comprising 20–30% of cases [1]. Rare causes of WS include renal infections, cystic diseases, nephrolithiasis, hypertension, diabetes, pregnancy, coagulation disorders, and anticoagulation therapy [1].

CKDs, especially ESRDs, are recognizable but rare causes for Wunderlich syndrome [6]. CKD is associated with a 1.5-fold increased bleeding risk compared with patients with normal kidney function [7]. Increased bleeding is thought to be driven principally by platelet dysfunction and an abnormal platelet–vessel wall interaction [8]. Platelets from uremic patients show abnormal adhesive function; reduced aggregating response to adenosine diphosphate, epinephrine, and collagen; and altered arachidonic acid metabolism [8]. Bleeding is multifaceted, and additional contributing factors have been described, such as alterations in the coagulation cascade, disrupted interactions between von Willebrand factor and platelet glycoprotein receptors, increased nitric oxide and prostacyclin, impaired fibrinolysis, and the sequalae of anemia. Anemia causes a tendency to bleed, probably due to the deranged radial transport of platelets, decreasing their contact with the endothelium [8].

The prevalence of abnormal bleeding increases as eGFR decreases [9]. Moreover, clinically relevant bleeding in CKD patients has been reported in 24–50% of patients on HD, with >2-fold increased prevalence compared with that in non-dialysis-dependent CKD patients [9]. The frequency of SRH is reported to be higher with HD than with peritoneal dialysis [2]. The incidence of WS in dialysis is 0,58%, with male predominance (74.5%) [2].

In individuals with ESRD, particularly those on hemodialysis, renal neoplasms (RCC and angiomyolipoma) and ACKD are the primary causes of WS [10]. ACKD is characterized by the development of multiple cortical cysts in atrophic kidneys. These cysts are fragile and prone to rupture, which can lead to spontaneous renal hemorrhage. The longer the duration of dialysis, the greater the likelihood of developing ACKD: ACKD develops in 10–20% of patients within the first 3 years of dialysis. By 5 years, 40–60% of patients develop ACKD, and after 10 years the incidence is >90% [10]. Several articles have described the association between SRH and previous development of ACKD [11,12,13]. Nonetheless, ACKD was not indicated in our patient.

Other authors have mentioned angiomyolipoma and RCC as primary causes of WS [14,15]. Moreover, ACKD is a well-established risk factor for the onset of RCC, with 80% of RCC cases linked to ACKD [16].

Dialysis-related amyloidosis (DRA), marked by the accumulation of β2-microglobulin amyloid, frequently occurs in patients undergoing long-term hemodialysis and is recognized as a possible predisposing condition for developing WS in this cohort of patients. DRA can be seen in as much as 20% of patients after 2–4 years of HD and in 100% of patients after 13 years of HD [17]. Amyloid angiopathy weakens renal vessel walls, increasing susceptibility to spontaneous rupture and perirenal hemorrhage, which can manifest clinically as WS.

In contrast, in our patient, regardless of the duration of hemodialysis therapy, neither pathological examination nor imaging studies showed evidence of ACKD, renal neoplasms, or DRA; therefore, we do not consider them causes of the WS in this case.

Chronic inflammation and oxidative stress associated with ESRD weaken renal tissues and vasculature, making the kidneys more susceptible to spontaneous bleeding [18]. The observed transverse renal lesion was therefore highly attributable to the compromised renal and parenchymal integrity and vascular fragility. Interestingly, histological examinations revealed not only nephrosclerosis, a frequent finding in patients with ESRD, but also signs of tubulointerstitial nephritis. CTIN affects the tubules, causing fibrosis, along with microvascular alterations and scar formation, which promote further fibrosis [19]. This may predispose the kidney to bleeding under stress or minor insults, though spontaneous large hematomas are rare without other contributing factors. Spontaneous subcapsular renal hematomas have been reported in hemodialysis patients with ESRD and often with interstitial fibrosis, sometimes related to small tumors or vascular fragility, but not directly to the CTIN itself [20].

Anticoagulation use, regardless of its indications, seems to be a factor for SRH [2]. Studies have reported instances of WS in individuals undergoing anticoagulation therapy, identifying anticoagulants as a contributing factor to SRH. Several case reports have described WS in patients on oral anticoagulants such as warfarin or direct oral anticoagulants (e.g., apixaban) [21,22]. However, these studies usually involve oral agents rather than heparin therapy alone.

Our patient received 0.4 LMWH-nadroparin solely during the dialysis session. Overall, the use of LMWHs has a similar or even lower risk of major bleeding compared with unfractionated heparin (UFN) in patients with renal insufficiency, including those on dialysis [23,24]. Among LMWHs, no significant difference in bleeding risk has been conclusively demonstrated, although enoxaparin may have higher anti-Xa peak levels [25]. Heparinization during dialysis can possibly explain the occurrence of SRH during or shortly after the hemodialysis sessions [2,3]. After subcutaneous administration, the half-life of nadroparin is between 3.5 h and 11.2 h in healthy subjects, contrary to dialysis patients, in which anticoagulant activity persists up to 40 h after the previous dialysis session [26]. However, our patient’s symptoms began a day before dialysis, more than two days after his last session, indicating no or insufficient circulation of heparin for hemorrhagic induction.

Although antiplatelet agents have also been reported in some cases, they are a less frequent cause of SRH [2]. A meta-analysis with 1131 hemodialysis patients revealed that these patients face an elevated risk of bleeding associated only with the administration of dual antiplatelet therapy. Furthermore, the use of a single antiplatelet agent did not appear to be associated with bleeding risk in the subgroup analysis [27]. Vascular access seems to be an additional reason for increased risk of bleeding, where dialysis catheters have been established to be linked to coagulation changes and higher bleeding risk [28]. Conversely, in our case, the vascular access was via the AVF.

In conclusion, we postulate that CKD and dialysis by themselves are a sufficient cause of the occurrence of Wunderlich syndrome due to the above-mentioned specific coagulation abnormalities and the impaired architectonics of the renal tissue, with the use of antithrombotic medications acting as an additional contributing factor.

Clinically, patients may present with isolated flank pain (67%), hematuria (40%), hypovolemic shock (27%), and Lenk’s triad (about 20%) [1]. SRH should be strongly suspected in every patient with ESRD (particularly those on hemodialysis) presenting with more or less severe abdominal or flank pain, hypotension, and a sudden-onset drop in hemoglobin [3].

Laboratory findings have included anemia and hematuria, and meta-analysis results have indicated that CT is 100% sensitive in detecting retroperitoneal hemorrhage and is superior to ultrasound for identifying underlying renal pathologies. Thus, CT remains the imaging modality of choice, allowing for precise measurement and monitoring of hematomas [29]. When a CT scan does not identify the underlying cause, angiography may be needed [30], as it is particularly useful when the CT findings are inconclusive and further investigation is required to identify the cause of the hematoma.

Initial reports suggested radical or modified nephrectomy for all cases. However, based on recent case series, conservative management may be a reasonable approach [16]. According to the European Association of Urology guideline, the management for renal trauma can be classified as non-operative (conservative and selective angioembolization) or operative [31]. The treatment approach depends on the patient’s hemodynamic stability, the extent of hemorrhage, and the presumed underlying pathology (Table 1) [1,31,32].

The recent data establish SAE as an effective and kidney-saving intervention in SRH patients [1]. However, gross hematuria, hemodynamic instability, and urinary extravasation are significant predictors of SAE failure [32].

Initially, our patient was managed conservatively given his stable condition. However, his condition deteriorated, with ongoing bleeding and hematoma enlargement. Considering the risks of the procedure and the lack of benefit in preserving the kidney, the patient underwent a right nephrectomy. Despite the life-threatening nature of WS, the patient achieved full recovery due to the expedited diagnosis and therapeutic management.

## 4. Conclusions

The presented case study underscores the importance of maintaining physicians’ high suspicion of spontaneous renal hemorrhage, as it presents a clinical and therapeutic dilemma, despite the rarity of this condition. Patients receiving dialysis therapy constitute a distinct high-risk group due to the number of risk factors associated with the dialysis itself, including paradoxical bleeding potential and disrupted kidney architectonics, as well as due to the contributing role of antithrombotic drugs. Early surgical intervention proved lifesaving in this instance, though it also highlights the challenges of managing catastrophic bleeding complications in this fragile population.

## Figures and Tables

**Table 1:** Therapeutic approach in patients with Wunderlich syndrome.

Types of Management	Indications
Non-operative Conservative Selective angioembolization (SAE)	• hemodynamically stable patients; • patients with benign disease; • without evidence of significant pathology; • active bleeding control in hemodynamically unstable patients and/or high surgical risk (Exclusively for SAE).
B. Operative	• hemodynamically unstable patients; • failure of non-operative treatment; • progressive anemia; • a palpable retroperitoneal hematoma; • symptoms of peritoneal irritation; • suspicion of malignancy

## Data Availability

The original contributions presented in this study are included in the article. Further inquiries can be directed to the corresponding author.
